# Editorial: Pathophysiology of bone and mineral metabolism

**DOI:** 10.3389/fphys.2024.1383660

**Published:** 2024-02-23

**Authors:** Elena Ambrogini, Amy Y. Sato, Manuel Naves-Diaz, Juan M. Díaz-Tocados

**Affiliations:** ^1^ John L. McClellan Memorial Veterans Hospital, Central Arkansas Veterans Healthcare System, Veterans Health Administration, United States Department of Veterans Affairs, Little Rock, AR, United States; ^2^ Center for Musculoskeletal Disease Research, University of Arkansas for Medical Sciences, Little Rock, AR, United States; ^3^ Department of Orthopedic Surgery, University of Arkansas for Medical Sciences, Little Rock, AR, United States; ^4^ Department of Physiology and Cell Biology, University of Arkansas for Medical Sciences, Little Rock, AR, United States; ^5^ Bone and Vascular Metabolism and Chronic Inflammatory Diseases, Health Research Institute of Asturias (ISPA), Oviedo, Spain; ^6^ Bone and Mineral Research Unit, Central University Hospital of Asturias, Oviedo, Spain; ^7^ Vascular and Renal Translational Research Group, Biomedical Research Institute of Lleida (IRBLleida), Lleida, Spain

**Keywords:** osteoporosis, axial spondyloarthritis (AxSpA), renal osteodystrophy, diabetes and obesity, primary hyperparathyroidism, mendelian randomization, antiresorptive treatments, serum bone markers

Bone plays a crucial role in maintaining overall systemic balance, but it is often affected by various chronic diseases and acute conditions, leading to bone mineral density loss. Aging, hormonal changes as well as factors like diet and physical activity also impact bone health. This vulnerability to multiple influences can reduce bone mineral density (BMD), increasing the risk of fractures, which are linked to higher mortality and cardiovascular disease. Treatments to prevent bone loss exist, including antiresorptive and osteoanabolic therapies. This Research Topic entitled “*Pathophysiology of Bone and Mineral Metabolism*” includes nine articles focusing on bone abnormalities in different diseases and treatments to prevent bone loss.

Wang et al. report the changes in serum calcium, vitamin D and parathyroid hormone (PTH) levels in women with pseudohypoparathyroidism (PHP), a rare disease affecting 1.2/100,000 people, during gestation and *postpartum*, when calcium metabolism is actually affected. The authors observed that high PTH levels could be predictors of a worsened calcium metabolism and treatment requirement and highlight the importance of controlling serum PTH concentration, calcium supplementation and calcitriol treatment during pregnancy to maintain normal calcium metabolism in PHP patients.

By Mendelian randomization analysis, Fu et al. studied the causal effects of blood homocysteine and serum levels of vitamins B6 and B12 on BMD at different bone sites and risk of fractures using public GWAS data from large-cohort studies. They found that the increased genetic prediction of homocysteine concentrations was causally associated with reduced heel BMD, and elevated genetic prediction of vitamin B12 had a causal impact on reduced total body BMD, especially at age over 45 years. However, there was no association of serum vitamins B6 and B12 or blood homocysteine concentrations with the risk of fracture. These observations encourage additional studies to clarify the existence of novel roles for B vitamins and homocysteine in the regulation of BMD and their use as potential treatments in the prevention of osteoporosis.

Another Mendelian randomization study conducted by Jiang et al. explores the causal association between BMD and osteoarthritis (OA) risk using data from a previous meta-analysis of total body BMD including 66,628 individuals and observed a causal association between higher genetic susceptibility to BMD and increased risk of OA, a link that can be of interest in clinical practices.

In a retrospective study, Wang et al. observed that patients with sepsis showed increased levels of serum markers of bone resorption as compared with non-sepsis controls. The authors also found higher concentrations piezo-type mechanosensitive ion channel component 1 (PIEZO1) in the serum of these patients, suggesting that circulating PIEZO1 could be associated with the inflammatory status in these patients. In addition, they suggest that serum levels of PIEZO1 together with concentration of markers of bone resorption may predict the occurrence of sepsis or sepsis sock, as well as increased mortality after 1 month.

In an interesting meta-analysis, Rajput et al. investigate the effects of two commonly used anti-resorptive therapies (bisphosphonates and denosumab), in the prevention of hypercalcemia and bone loss in primary hyperparathyroidism (PHPT). Their results showed that treatment with bisphosphonates and denosumab or denosumab alone results in higher BMD at the lumbar spine and femoral neck after 1 year, while bisphosphonate use only increased BMD at lumbar spine. All anti-resorptive treatments resulted in lower serum calcium concentration, increased serum PTH levels and lower serum concentration of markers of bone formation in PHPT patients, indicating that strong suppression of bone resorption by anti-resorptive therapy in PHPT may lead to the exacerbation of hyperparathyroidism despite of the improvement in BMD.

In a cross-sectional analysis including 10,564 elderly participants, Hou et al. studied the association between smoking status and serum levels of cotinine, a metabolite of nicotine, with bone loss (osteoporosis or osteopenia). They found that higher serum cotinine levels or being current smoker associated with higher prevalence of osteoporosis in three multivariate logistic regression models. Moreover, the authors demonstrated a positive correlation of serum cotinine levels and high prevalence of osteoporosis and osteopenia, suggesting possible mechanisms involving tobacco exposure in bone loss.

In an interesting study, Gómez-García et al. investigated the distinct bone and inflammatory profile of axial spondyloarthritis patients with or without radiographic features (r-axSpA or nr-axSpA respectively). They found that patients with r-axSpA have low plasma levels of bone formation markers as compared with controls, accompanied by low sclerostin concentration and higher levels of pro-inflammatory molecules. Notably, nr-axSpA patients had higher serum levels of the anti-inflammatory cytokine Interleukin 13 in comparison with r-axSpA patients and controls, which could exert protective effects on disease progression.

This Research Topic also includes two mini reviews, both focused on active areas of research in bone disease: On one hand, Aguilar et al. summarized current knowledge on the bone and mineral complications associated with chronic kidney disease (CKD-MBD), one of the most important factors increasing hip fracture occurrence together with aging. They address important Research Topic such as the need of performing a bone biopsy, factors that may affect diagnosis of bone disorders, the detrimental effects of secondary hyperparathyroidism and the mechanisms involved in bone alterations, as well as the use of current anti-osteoporotic treatments in CKD. On the other hand, Bathina and Armamento-Villareal revised the mechanisms involved in the imbalance of osteogenic-adipogenic differentiation in type 2 diabetes (T2DM) and obesity at cellular level, such as inflammation and oxidative stress. It is important to note that both, T2DM and obesity, are associated with an increased risk of fractures, highlighting the role of bone as an orchestrator of metabolic homeostasis. Furthermore, in this comprehensive review, the authors discuss the effectiveness of current treatments and drug repurposing on increasing bone formation in T2DM and obesity patients.

